# Geographic information systems and logistic regression for high-resolution malaria risk mapping in a rural settlement of the southern Brazilian Amazon

**DOI:** 10.1186/1475-2875-12-420

**Published:** 2013-11-15

**Authors:** Elaine Cristina de Oliveira, Emerson Soares dos Santos, Peter Zeilhofer, Reinaldo Souza-Santos, Marina Atanaka-Santos

**Affiliations:** 1 Epidemiological Surveillance, Health Secretary of Mato Grosso, Rua D, Political Administrative Center, Cuiabá, Mato Grosso State 78.050-970, Brazil; 2 Department of Geography, Federal University of Mato Grosso, Av. Fernando Corrêa, Cuiabá, Mato Grosso State 78.060-900, Brazil; 3 Department of Endemic Disease, Brazilian National School of Public Health, Oswaldo Cruz Foundation, Rua Leopoldo Bulhões, 1480, Rio de Janeiro, Rio de Janeiro State 21.041-210, Brazil; 4 Institute of Public Health, Federal University of Mato Grosso, Av. Fernando Corrêa, Cuiabá, Mato Grosso State 78.060-900, Brazil

**Keywords:** Malaria, Remote sensing, Spatial analysis, Epidemiology

## Abstract

**Background:**

In Brazil, 99% of the cases of malaria are concentrated in the Amazon region, with high level of transmission. The objectives of the study were to use geographic information systems (GIS) analysis and logistic regression as a tool to identify and analyse the relative likelihood and its socio-environmental determinants of malaria infection in the Vale do Amanhecer rural settlement, Brazil.

**Methods:**

A GIS database of georeferenced malaria cases, recorded in 2005, and multiple explanatory data layers was built, based on a multispectral Landsat 5 TM image, digital map of the settlement blocks and a SRTM digital elevation model. Satellite imagery was used to map the spatial patterns of land use and cover (LUC) and to derive spectral indices of vegetation density (NDVI) and soil/vegetation humidity (VSHI). An Euclidian distance operator was applied to measure proximity of domiciles to potential mosquito breeding habitats and gold mining areas. The malaria risk model was generated by multiple logistic regression, in which environmental factors were considered as independent variables and the number of cases, binarized by a threshold value was the dependent variable.

**Results:**

Out of a total of 336 cases of malaria, 133 positive slides were from inhabitants at Road 08, which corresponds to 37.60% of the notifications. The southern region of the settlement presented 276 cases and a greater number of domiciles in which more than ten cases/home were notified. From these, 102 (30.36%) cases were caused by *Plasmodium falciparum* and 174 (51.79%) cases by *Plasmodium vivax.* Malaria risk is the highest in the south of the settlement, associated with proximity to gold mining sites, intense land use, high levels of soil/vegetation humidity and low vegetation density.

**Conclusions:**

Mid-resolution, remote sensing data and GIS-derived distance measures can be successfully combined with digital maps of the housing location of (non-) infected inhabitants to predict relative likelihood of disease infection through the analysis by logistic regression. Obtained findings on the relation between malaria cases and environmental factors should be applied in the future for land use planning in rural settlements in the Southern Amazon to minimize risks of disease transmission.

## Background

Malaria is one a major public health problem and it affects more than three hundred million individuals per year. It severely impacts the African continent and affects more than one million people per year in the Amazon countries in South America. Brazil accounts for one-third of the reported malaria cases [1].

Malaria is one of the most serious and striking of the transmissible diseases, and it affects approximately 500 million people per year worldwide, causing more than one million deaths each year [2]. In Brazil, 99% of the cases of malaria are concentrated in the Amazon region. The disease has been highly transmissible, maintaining high levels that are superior to those of the 1970s, when 3.9 cases per 1,000 inhabitants in the Amazon region were recorded. In 1999, 2001 and 2003, the annual parasite index (API) (number of positive slides for malaria/year per 1,000 inhabitants) of the region was of 31.9, 18.8 and 19.3 cases per 1,000 inhabitants, respectively [3]. In 2005, 90% of the cases of malaria were registered from the recent occupation of rural areas, with activities such as manual gold mining, wood extraction and subsistence crop and cattle farming. On a regional scale, the distribution of malaria is commonly associated with environmental conditions and, mainly, with the tropical climate [4].

In 2005, Mato Grosso registered 9,774 cases of malaria, which corresponded to 2% of the total of cases in the Legal Amazon. When compared to 2004 (7,849 cases), there was a 38.7% increase of the cases in the State. The cities of Juruena and Rondolândia nearby in the northwest of the State deserve special attention for their high malaria incidences with APIs higher than 50 per 1.000 inhabitants [5]. Climatic conditions guarantee the reproduction and longevity of the *Anopheles* mosquitoes that transmit the disease. Environmental factors, such as the periodicity of rainfall and the flood and recession of the Amazon tributaries, paired with social aspects such as the extension of the disorganized land occupation and installation of gold mining sites, are factors that have favoured and been responsible for the occurrences of malaria in the cities in the north of the State [6].

The city of Juruena presented 720 positive smears for malaria in 2004. This corresponded to an API of 116.8 per 1.000 inhabitants, which represented an increase of 284.9% in the incidence of positive slides when compared to the 2003 API of 41.0 per 1,000 inhabitants [7].

**Figure 1:**
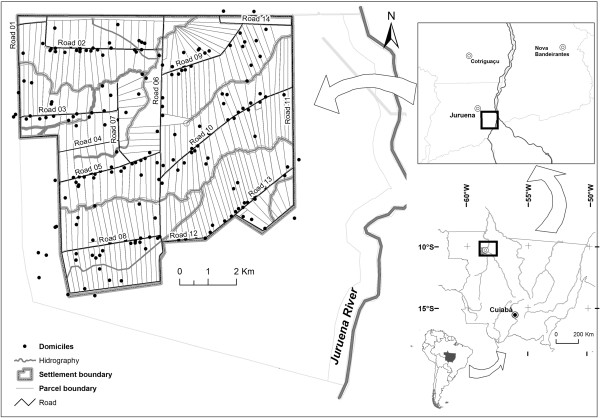
Geographical location and spatial configuration of road network and domiciles in the Vale do Amanhecer rural settlement in Brazil.

The studied Vale do Amanhecer rural settlement is located in the northwest of the State of Mato Grosso, about 800 km northwest from the capital Cuiabá, 10°26′47″ and 10°21′46″ southern latitude; 58°28′16″ and 58°22′39″western longitude inside the municipality of Juruena, 6.2 km southeast from the city centre. The Vale do Amanhecer Settlement Project was created by the National Institute of Colonization and Agrarian Reform [8] in 1998. It has an area of 14,400 ha, of which 7,200 ha were reserved for the occupation of a maximum 250 families. Another area of 7,200 ha is designated as a natural permanent reserve. The parcels have 26 ha each and are distributed in lines along projected roads, numbered from 01 to 14 (Figure 1).

As it is part of the Amazon biome, this area originally had a predominance of semi-evergreen seasonal and evergreen forests with trees as tall as 50 m, and is located on a plateau with moderately hilly terrain in altitudes between about 205 and 295 m. The tropical lowland rainforest climate is characterized by an annual mean temperature of about 27°C and precipitations of 2,250 mm per year with two distinct periods; about 80% of the rainfall occurs between September and April, whereas monthly precipitation from May to August is mostly lower than 50 mm.

Remote sensing (RS) and geographic information systems (GIS) have proved to be an innovative and important component in studies of public health and epidemiology [9] and have been used for monitoring, surveillance and spatial modelling of diseases, provide examples of how Eearth observation satellites can be used in studies of ecology and prediction of malaria, and have contributed with examples for the mapping of malaria vectors, using mid-resolution RS-imagery such as Landsat ETM or SPOT [10-15].

In local-scale studies it has become common to use spatial analysis techniques, which allow the precise localization of risk areas, such as the distance between the vector’s breeding places and households, the dispersion of the vectors and case clusters [16-19].

Inasmuch as the risk of contracting malaria is related to diverse factors such as environmental alterations caused by human activities, this study aims to identify and analyse local-scale, spatial patterns of the disease in the Vale do Amanhecer settlement, an area with documented, elevated malaria incidences [20].

## Methods

### Data collection and analysis

**Figure 2:**
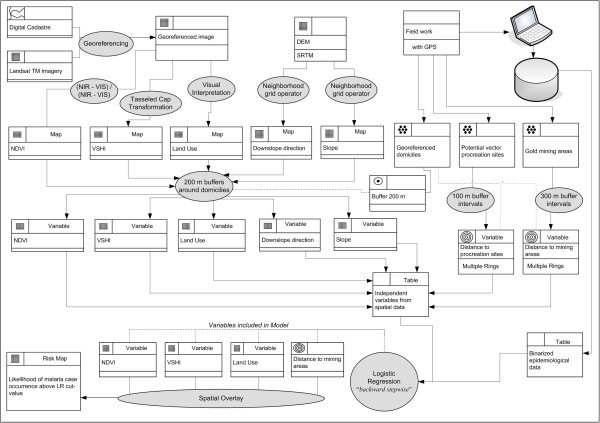
Methodological approach for malaria risk mapping in the Vale do Amanhecer rural settlement in Brazil.

Land use and cover, vegetation density, terrain slope and downslope direction, as well as the distance of domiciles from possible breeding grounds and gold mining sites were considered as potential independent variables to explain risk levels. GIS analysis and logistic regression (LR) were used for spatial risk modelling (Figure 2).

### Epidemiological data

The record of 585 malaria cases for 2005 were obtained at the Municipal Secretary of Health of Juruena. Out of the total of notifications in the Juruena municipality, 336 came from the Vale do Amanhecer land reform settlement. Notification forms recording positive slides for malaria were compared to those recorded in the national Epidemiological Surveillance Information System for malaria [21]. Comparisons were made in order to verify data consistency as well as to complement the data when fields in the photocopied forms were illegible. The EpiInfo 3.3.2 (CDC) software was used to build the spatial database of malaria cases. Notification forms of positive slides were related to individuals domiciles, georeferenced in geographical coordinates by a GPS field survey (SAD 69 Datum). The database was then exported to the TerraView 3.1.4 (INPE) GIS software.

### Spatial data processing

SPRING 4.2 (INPE) and ENVI (RSI) were applied for satellite imagery pre-processing such as band composition and calculation of spectral indices such as the normalized difference vegetation index (NDVI) and the soil and vegetation humidity index (VSHI) of the Tasseled Cap Transformation. Georeferencing and processing of vector layers, such as georeferenced field data and the settlement digital map (1:50.000 scale) obtained from INCRA (National Institute for Colonization and Agrarian Reform), visual interpretation for land-use mapping and spatial analysis procedures such as buffering were realized in the ArcGIS 9.2 (ESRI) and TerraView 3.1.4 (INPE).

Land Use and Cover (LUC) mapping was realized using a multispectral Landsat 5 TM-image provided by the Instituto Nacional de Pesquisas Espaciais (INPE) from 25/06/2005 (WRS 229/67).Visual interpretation of Landsat TM imagery by experts generally outperforms supervised classification techniques [22,23]. Therefore the LUC map was elaborated by visual interpretation of a #3/#4/#5 colour composite, resulting in three classes coded ordinally to 1 - forest, 2 - secondary vegetation and 3 - agricultural area.

The NDVI was scaled into 256 grey levels and then three classes defined: low (0 to 100), intermediate (101 to 200) and high (> 200). The soil and vegetation humidity index (VSHI) was obtained as the third component of a Tasseled Cap Transformation [24], which represents the continuous distribution of the wetness of vegetation/soil during satellite passage (dry season) and then differentiated into three equally spaced classes of the dynamic range of the VSHI scene.

Slope and downslope direction maps, proxies for terrain shadowing and therefore potentially relevant for vector habitat sutiability [25,26], were extracted from a 90 m resolution digital elevation model of the Shuttle Radar Topography Mission (SRTM). The slope map represents the continuous distribution of the terrain slope with values ranging from 0 to 16 degrees. The downslope direction map is a representation of nine classes of slope direction (eight main directions and “flat”), which were used in the logistic regression model as nominal coded.

Distance of domiciles to potential breeding sites [27-29] and mining areas [30,31] has shown to have potential predictive power in household malaria risk mapping. Euclidian distance maps to water bodies with 100 m intervals, and to mining areas with 300 m intervals were generated using standard buffering techniques. These different intervals were chosen because of their maximum distances to the domiciles, ranging from 2,900 m at maximum in the case of most distant potential breeding site, and 6,900 m in the case of the most distant mining area.

### Ethical considerations

This study is part of the malaria spatial analysis in rural land reform settlements project and was approved by the Julio Muller Hospital Ethics Committee, protocol number 326/CEP/HUJM/07 on the 9 May, 2007. This study is based on secondary data made available by the Health Sector of the Municipalities included in the investigation. The data banks do not include names or other information that allow for identifying the subjects.

### Data analysis and spatial modelling

The model that represents the likelihood of infection by malaria at the Vale do Amanhecer settlement was generated based on Logistic Regression with the ‘backward conditional stepwise’ procedure, comparing cases/non-cases with multiple explanatory variables. It was opted for a dichotomous modeling approach, as no reliable statics on the total number of inhabitants domicile could be obtained, which would a pre-requisite of bias-free estimate of absolute cases or its probability (eg. by Poisson regression or generalized linear models). Model performance for different cut-off values was assessed by its sensitivity and specificity (ROC curve). In logistic regression, the canonical link function (logits) for the binomial distribution, of the unknown binomial probabilities are modelled as a linear function of the risk factors (x_i_):

$$g\left(P_{i}\right)={ß}_{0}+{ß}_{1}x_{1}+{ß}_{2}x_{2}+\cdots +{ß}_{i}x_{i}$$

In which:

*g(P*_*i*_*)* = link function

*P*_*i*_ = likelihood of response for the –ith factor (or covariate)

β_*0*_ = intercept

β_*i*_ = coefficient

*x*_*i*_ = independent variables

Logistic regression outcomes such as the Wald statistics, significance levels of variable coefficients and overall classification accuracy were used to test the importance of the environmental factors for the occurrence of malaria cases and for the development of a risk model. Using the stepwise, backward conditional method, as implemented in SPSS 15 (SPSS Inc.), only variables with significance higher than 95% (p < 0.05) were maintained in the final model (Table 1).

**Table 1:** Exploratory variables for the analysis used in the logistic regression for malaria infection model in rural settlement in Brazilian Amazon

**Variable**	**Scale**	**Number of classes**
Land Use	Nominal	3
Vegetation index (NDVI)	Ordinal	3
Vegetation and soil humidity index (VSHI)	Ordinal	3
Procreation distance	Absolute	Continuous
Mining area distance	Absolute	Continuous
Slope	Absolute	Continuous
Downslope direction	Nominal	9

## Results

In 2005, the Vale do Amanhecer settlement had 718 inhabitants, of which 394 (54.87%) were men and 324 (45.13%) were women. In the settlement, 359 cases of malaria were notified, distributed in 200 domiciles, the city of Juruena presented 720 positive smears for malaria in 2004. This corresponded to an API of 116.8 per 1.000 inhabitants, which represented an increase of 284.9% in the incidence of positive slides when compared to the 2003 API of 41.0 per 1,000 inhabitants [7].

Five cases were excluded because the notification forms of SIVEP-malaria did not inform their dwelling places, and another 18 because they belonged to domiciles outside the settlement limits. The 7,200 ha reserved for land owners and another 7,200 ha designated to permanent environmental reserve were considered the official limit area of the settlement.

**Figure 3:**
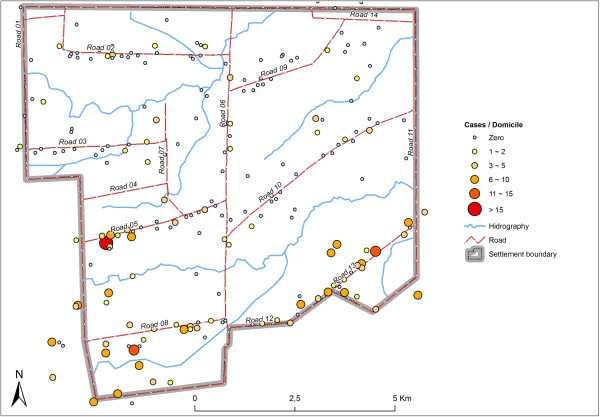
Distribution of malaria cases in and near the rural settlement in Brazilian Amazon, considered for logistic regression model building.

Out of the total 336 cases of malaria, 133 positive slides were from dwellers at Road 08, which corresponds to 37.60% of the notifications. As for Roads 13 and 5, 124 cases were notified (35, 10%) and 58 (16, 40%) cases of malaria were notified, respectively (Figure 3).

**Figure 4:**
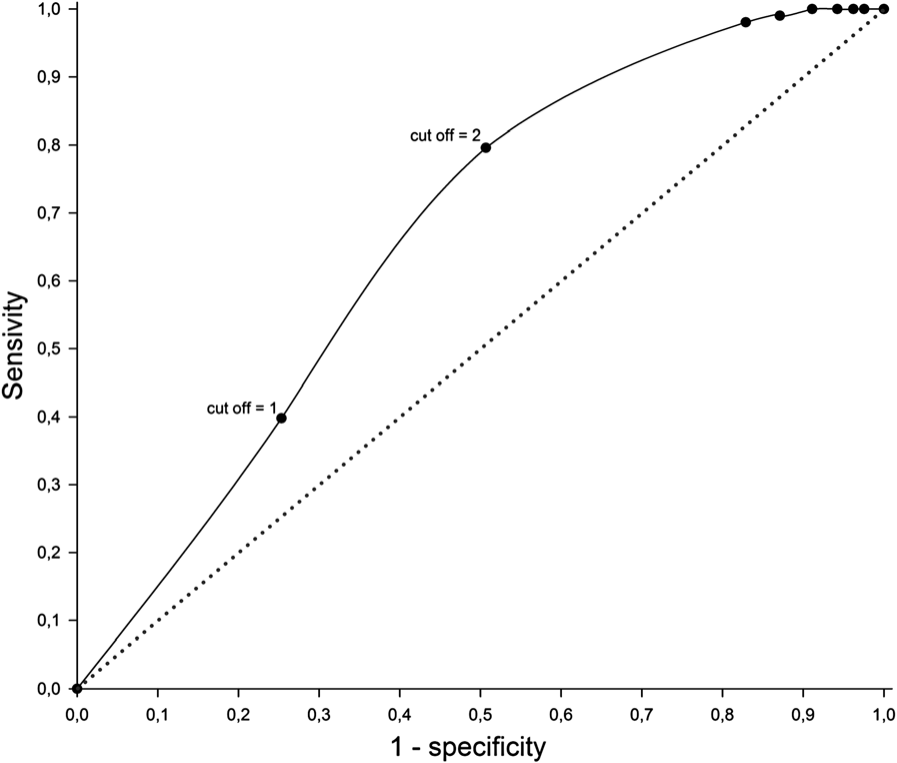
ROC curve of the cases of malaria cut-off values.

The logistic regression model performance for a cut-value of at least one case per domicile show a poor overall performance (65.4%), very low sensitivity (0.39) and percentage of explained variance (Nagelkerke R square = 0.22), as only VSHI and mining area distance are found to be significant predictors (Figure 4). A cut-value of 2 strongly improves the logistic regression model, increasing overall performance to 74.5%, sensitivity to 0.79 and Nagelkerke R square to 0.46. For higher cut-offs, both overall performance and specificity sharply decrease. This model for a cut-off value of 2 included as significant (p < 0.05) the variables Land Use, VSHI, and NDVI; moreover, it included as highly significant (p < 0.01) the variable mining area distance (Table 2).

**Table 2:** Parameters of variables included in the logistic regression model for malaria infection in the rural settlement in Brazilian Amazon

**Step**	**Variables**	**Coefficient (ß)**	**S.E.**	**Wald**	**Sig. (p)**	**Exp (B)**
**1**	Land Use	0.554	0.286	3.738	0.053	1.740
	Slope	0.072	0.085	0.713	0.399	1.075
	Downslope direction*	0.024	0.089	0.070	0.792	1.024
	VSHI	0.009	0.005	3.545	0.060	1.009
	NDVI	−0.014	0.006	4.803	0.028	0.986
	Mining area distance	−0.481	0.176	7.475	0.006	0.618
	Procreation distance	0.133	0.196	0.459	0.498	1.142
**2**	Land Use	0.561	0.285	3.862	0.049	1.752
	Slope	0.077	0.084	0.835	0.361	1.080
	VSHI	0.010	0.005	3.764	0.052	1.010
	NDVI	−0.014	0.006	4.852	0.028	0.986
	Mining area distance	−0.471	0.172	7.509	0.006	0.624
	Procreation distance*	0.131	0.196	0.450	0.502	1.140
**3**	Land Use	0.550	0.282	3.795	0.051	1.734
	Slope*	0.090	0.081	1.233	0.267	1.094
	VSHI	0.010	0.005	4.420	0.236	1.010
	NDVI	−0.014	0.006	5.248	0.022	0.986
	Mining area distance	−0.371	0.081	20.725	0.000	0.690
**4**	Land Use	0.533	0.280	3.626	0.047	1.704
	VSHI	0.011	0.005	5.478	0.019	1.011
	NDVI	−0.015	0.006	5.813	0.016	0.985
	Mining area distance	−0.312	0.061	26.408	0.000	0.732

*Variables removed during stepwise procedure.

**Figure 5:**
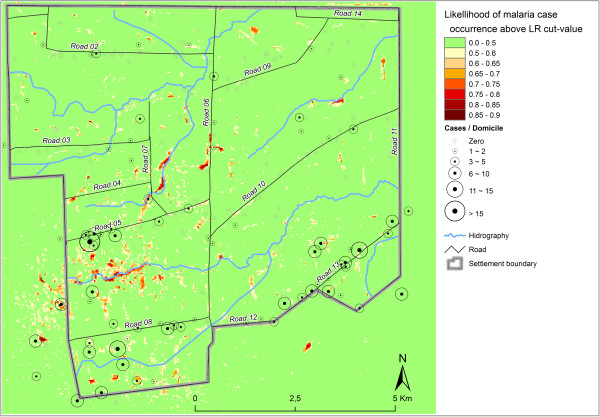
Relative risk of malaria infection in the Vale do Amanhecer settlement, Juruena, MT, 2005.

The Wald statistics underpin the high importance of this variable (26.4) as predictor. Consequently, areas with highest likelihoods of malaria infection are located in the southern part of the settlement, where mining activities are concentrated (negative variable coefficient). In the model these situations are related. The highest relative likelihoods however are only obtained if an area presents intense use and occupation, high level of wetness (positive coefficients) and low NDVIs, indicating little remaining vegetation (Table 2). Additional hot-spots of elevated risks occur in the mid-western region of the settlement (Figure 5) where secondary predictors such as VSHI, NDVI and land use account for elevated risks, but where the density of mining areas is lower.

## Discussion

Malaria risk and its predictive mapping in South America should be addressed under four interconnected spatial scales, which have to be considered even if a study focuses on a local scale. First, continental scale, high temperature, humid and semi-humid tropical, lowland climates favour elevated vector densities and malaria transmission in the Southern Amazon [32,33]; second, (super) regional scale, which is little discussed in the literature yet, inside this climatic zone, extreme spatial heterogenities in malaria cases can be observed, ranging from less than 0.5 malaria incidences per 1,000 inhabitants in municipalities with consolidated land use, against up to more than 420 incidences in 2005 in municipalities with mining activities and/or land reform settlements [1], such as Juruena, where rural population densities are commonly higher than in areas with large crop farming activity. Inside high incidence municipalities, cases are further strongly clustered, frequently in areas with selective logging and mining activities [34,35]. Mining areas in Mato Grosso have been found to have high rates of asymptomatic infection making disease control difficult [36]. The assessment showed that malaria incidence in the Vale de Amanhecer settlement is more than ten times higher than in the rest of the municipality, probable favoured by an intense flux of populations with low immunity, poor housing conditions and precarious health care [36-39]. Due to the very high incidences in the settlement, logistic regression modeling results only in a reasonable overall performance, explained variance and sensitivity, if binarization is increased from one to two cases per household.

As reported by Rodrigues [40], the present study gives further evidence that GIS and logistic regression can be successfully applied for further local-scale zoning of high-risk areas. Greater likelihood of contracting malaria are significantly linked to areas close to gold mining sites [41], which present intense land use and occupation, high level in the VSHI and low NDVI values. That high incidences are related to the fine-grained spatial association between natural preserved environments of the vector habitat altering with heavily modified man-made landscape patches, characterized by the high exposition and abundant vector presence. In this context, emphasize that the positive association between malaria incidence in the Amazon with deforestation [42,43] cannot be generalized, but may sharply differ or even be inverted as a function of observation scale and socio-environmental covariates. There is no evidence that malaria risk is elevated because of the presence of larger deforestation patches inside the settlement *per se*, but because these areas are characterized by elevated population fluxes and are frequently mining areas with man-made reproduction habitats favouring vector presence.

As deforestation tends to be higher near households, the output map must be interpreted as the potential risk, considering the spatial distribution of housings, which means that the geographical pattern of relative likelihood of disease infection would be altered if housing patterns were different (e g, allocation of new families). The relative importance of socio-environmental factors as determined by the logistic regression model however, is not biased as cases and non-cases are related to households and their surroundings.

Some local-scale malaria studies have reported a positive association between malaria incidences and proximity to watercourses [44], a variable not included as significant predictor in the logistic regression model in the present study. That these results have two reasons, mainly. First, most housing is located near watercourses, therefore, distance has a low amplitude of variation, and high distance, which hypothetically could have more non-cases, is under-represented. Second, mining areas are located along watercourses to facilitate gold extraction and include a mosaic of small man-made waterbodies. Therefore, both distance to watercourses and potential reproduction habitats have highly significant rank correlation with distance to mining areas and are therefore omitted as predictors in the final model.

Similarly, attribute lack of significant relationship of malaria incidences with DEM derived variables such as “slope” and “downslope” direction [25], with the spatial scale and the geographical configuration of study area. The terrain in the settlement is mostly flat or slightly undulated, so differences related to relief shadowing are low if compared to relief variations in the region on a coarser scale. Finally, the minimum mapping unit of TM imagery on the order of 0.5–1.0 ha, appropriate for thematic mapping not finer than 1:50,000 (Federal Geographic Data Committee, 1992, Wright *et al*. 2007, Collins and Stephens 2010), may explain the limited predictive power of land-use in the final logistic regression model.

## Conclusions

Malaria is a focal disease, and even this small settlement area presented heterogeneity in the spatial distribution of incidences. These patterns are less related to the natural environment *per se*, than caused by land use, landscape modification due to human activities in the settlement and the proximity of individuals to places with elevated vector presence.

In a high malaria risk area, GIS and logistic regression could be successfully applied to predict relative likelihood of disease infection, which is positively related principally to proximity of gold mining areas and elevated nearby mining areas and, secondarily, to intense land use, lower vegetation density and higher soil humidity.

Findings on the relationship between malaria cases and environmental factors should be applied in the future for land use planning in rural settlements in the Southern Amazon to minimize risks of disease transmission.

## Competing interests

The authors declare that they have no competing interests.

## Authors’ contributions

ECO and ESS contributed in the methodological study design, data acquisition, processing and analysis and manuscript preparation. RSS, MAS, PZ, and ESS participated in the study conception, scientific coordination and revision of the manuscript. All authors read and approved the final manuscript.
